# Une visite chez le coiffeur

**DOI:** 10.11604/pamj.2013.15.113.2980

**Published:** 2013-07-28

**Authors:** Fatima Zahra Hajji Ouafi, Hassam Badreddine

**Affiliations:** 1 Service de dermatologie CHU Ibn Sina Rabat, Maroc

**Keywords:** Tumeur noire, cuir chevelu, mélanome nodulaire

## Image en médicine

Patient âgé de 64 ans, ancien agriculteur, sans antécédents pathologiques particuliers, présente depuis février 2011, une plaque pigmentée, découverte par son coiffeur, qui a rapidement augmenté de taille, qui s'est surmontée de nodules dont un s'est ulcéré et saignait au contact, associée à des douleurs en casque sans notion de troubles digestifs, ou de douleurs osseuses. Le patient a bénéficié de consultations antérieures mis sous traitement non précisé sans amélioration. A l'examen clinique, il présente une plaque pigmentée surmontée de nodules et ulcérée par endroit d'environ 10cm/6cm avec des multiples nodules de perméation. L'examen des aires ganglionnaires retrouve une adénopathie latéro-cervicale droite d'1cm non inflammatoire et une 2ème sus-claviculaire homolatérale. Le reste de l'examen somatique était sans particularités. En dermoscopie, on retrouve un voile bleu-gris, de multiples globules et tâches bleues-gris. Les diagnostics évoqués sont: mélanome nodulaire, carcinome basocellulaire pigmenté, kératose séborrhéique, un angiome thrombosé. Dans ce sens, une biopsie cutanée a été réalisée et qui est en faveur d'un mélanome nodulaire, le niveau de Clark et de Breslow étaient difficiles à préciser étant donné l'envahissement total de la lésion. L'immunohistochinmie réalisée montre un HMB45 positif, et un KL1 négatif. Le bilan d'extension retrouve de multiples nodules parenchymateux pulmonaires au scanner des 4 étages. La biopsie ganglionnaire d'une ADP sus claviculaire est en faveur d'une métastase ganglionnaire massive d'un mélanome. Il s'agit d'un mélanome métastatique stade IV, une chimiothérapie palliative type “Dacarbazine” a été indiquée.

**Figure 1:**
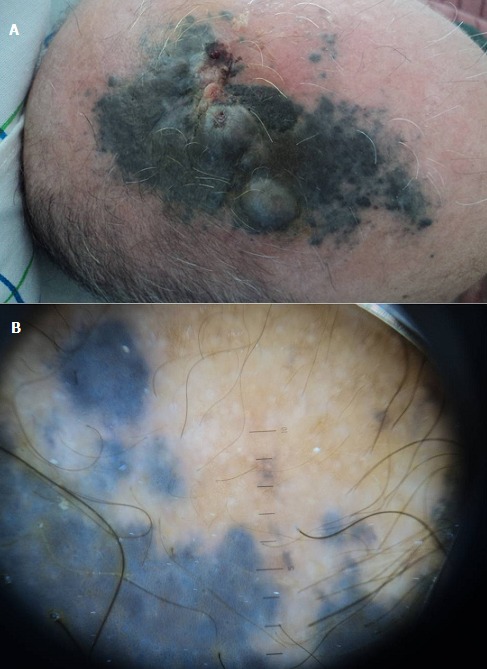
A) Mélanome nodulaire du cuir chevelu; B) Image dermoscopique

